# Prevalence and Psychosocial Correlates of After-School Activities among Chinese Adolescents in Hong Kong

**DOI:** 10.3389/fpubh.2014.00159

**Published:** 2014-09-25

**Authors:** Cecilia M. S. Ma, Daniel Tan Lei Shek

**Affiliations:** ^1^The Hong Kong Polytechnic University, Hong Kong, China

**Keywords:** after-school activities, positive youth development, Chinese adolescents, family functioning, Hong Kong

## Abstract

Using a cross-sectional design, this study (a) explores the prevalence of after-school activities among Chinese early adolescents and (b) assesses the relationships between participation in after-school activities, personal well-being, and family functioning. A total of 3,328 Grade 7 students (mean age = 12.59 years, SD = 0.74) completed a self-administered questionnaire. Results showed that the majority of adolescents returned home under adult supervision. Further analyses showed the associations between after-school activities, positive youth development qualities, academic and school competence, family functioning, and risky behavior. Implications regarding efforts aimed at designing high quality and structured after-school youth programs are discussed.

## Introduction

How adolescents spend their time after the school has been examined in the past decades. Generally speaking, previous cross-sectional studies have demonstrated the positive influence of after-school activities as well as experiences and youth development outcomes, such as school outcomes, social adjustment, civic engagement, and problem behaviors (1–3). The developmental significance of after-school time activities is further supported in longitudinal research. For instance, Mueller et al. (4) used a longitudinal design to study the relationship between participation in youth program and developmental outcomes (e.g., self-regulation skills and contribution to the society) among eighth to tenth graders. Results showed that adolescents’ participation in after-school program predicted their intention to contribute to the family and community after 2 years. Based on three waves of data, Kort-Butler and Hagewen (5) found that the type of extracurricular activity portfolio affected the trajectories of self-esteem from mid-adolescence to young adulthood.

Despite the growing body of research showing the positive effect of after-school activities on youth development, there are several research gaps in this field. First, existing findings on the relations between after-school experience and adolescents’ socio-emotional development failed to assess whether the results vary according to the nature of the activity. Existing studies have mainly confined to the influence of structured activities, such as organized sports, extracurricular activities, and religious groups, which are commonly supervised by adults and governed by rules and goals. Research has shown that adolescents who participated in structured activities showed higher levels of social trust (6), self-esteem (7), decision making skills (8), and lower levels of delinquent behaviors (9) than those who did not participate.

In contrast, little is known about the influence of unstructured activities on adolescents’ well-being (e.g., life satisfaction) and perceived school and academic competencies. Unlike structured activities, these activities are often initiated by the participants without adult supervision (e.g., “hanging out”, playing, reading, and surfing the internet) (10–12). Larson (13, 14) argued that these unstructured activities might also contribute to positive youth development due to its intrinsically motivated and voluntary nature, and thereby promoting open and uncritical peer interactions. This activity might also promote “secondary socialization” that helps youth to be socialized into group norms and membership (14). Unlike the adult-directed activities, children learn how to make their own decisions, solve their own problems, regulate their emotions and get along with friends, and follow rules and norms in the unsupervised context (15). The practice of self-control not only serves as a source of pleasure but also allows children to develop competence and confidence when problems arise (15, 16). These activities provide them, who act as “organizers of development” (17, 18), a unique context to develop and equip various kinds of positive competencies and skills (14).

Second, as prior research on after-school activity primarily focuses on reducing problem behaviors (9, 19), researchers have advocated a strength-based approach for promoting positive outcomes among adolescents (14, 20). Studies showed that participation in structured after-school activities was associated with prosocial behavior (21), self-regulation (4), life satisfaction (22), self-esteem (23) and related to fewer depressive symptoms, lower levels of anxiety (24), and less likely to engage in adolescent problem behaviors, such as pornography consumption (25, 26), substance use (27), delinquency (28), and internet addiction (29). More research is warranted to explore whether the benefits of participation in after-school activities exist in different dimensions of positive youth development qualities. Given the impact on positive youth development outcomes remains largely unexplored, the current study will examine the relationships between after-school activity and various positive youth developmental outcomes among early Chinese adolescents in Hong Kong.

Third, as most studies are conducted in Western contexts, it is not clear whether the benefits of after-school time activity also exist in the Chinese context. Under the influence of Confucianism, Chinese children are encouraged to study at the expense of leisure activity, which is in contrast to the beliefs in the Western culture that leisure activities and play are good ways to reduce physical and psychological stress. Chinese children grow up in a context where diligence is deeply cherished. They believe education as the only way of upward social mobility. They learn to work hard and make maximum use of time for study. This can be reflected in a Chinese proverb “Diligence has its reward; play as no advantages” (*qin you gong, xi wu yi*). The desire to excel academically is further supported by a study of at least 80% of middle school Chinese students spent over 8 h at school, which was longer than their parents spend at work (30).

Under the ever-increasing academic pressure, Chinese children have to do homework and prepare for examinations until late at night. Facing the competitive academic atmosphere and limited university places, Chinese children, especially those from affluent families, are asked to enroll in extracurricular tutorial classes after school and during weekend. Three major stressors were reported by Chinese students when facing such pressure, including “over-scheduling,” “insufficient sleep,” and “little time for exercise” (30, 31). Researchers advocated the adoption of the developmental-ecological approach to understand the nature of developmental process in a given context (32, 33). This information “helps identifying individuals’ preparedness to engage in specific developmental process…how might other factors such as age, personality, culture, gender, and SES shape initial dispositions, youths experience and participate in an activity” [(15), p. 179].

The lack of empirical studies in Chinese contexts is further supported when doing literature search in electronic databases. A computer search of PsycINFO database in August 2014 using “after-school time” and “after-school hours” showed that there were 9 and 4 citations, respectively. These figures clearly represented the needs to study the impact of after-school time activity on Chinese adolescents’ well-being. Given that after-school activity provides a unique arena for structuring adolescent development (5), this paper, therefore, attempts to fill the research gap by assessing the relations of after-school activity and adolescents’ outcomes in a non-Western city, Hong Kong.

To date, with an exception of a few studies (34, 35), studies on the pattern of after-school activities among Chinese adolescents is scarce. Based on a longitudinal design, Shek (35) found that the level of after-school activity without adult supervision increased from 14.5 to 26.9% over 3 years among 2,559 Chinese early adolescents (Grade 7). In particular, he found that adolescents’ after-school time use was a significant factor in predicting of parent–child relational qualities and their psychological well-being. A limitation of this study was the inability to measure differences in developmental outcomes as a function of nature of after-school activity. Previous research also shows that the relation of after-school activity involvement and problem behaviors varied by activity structure (36). Results indicated that a positive association between participating in unstructured and unsupervised activities and deviant behavior (19, 36, 37). Although prior research (35) underscored the relationship between after-school activity, family (e.g., parenting style and parent-adolescent relationship) and individual outcomes (e.g., psychological well-being), the nature of activity, particularly unstructured activity (e.g., wandering street alone), was not tested.

Theoretically, it is expected that the associations differ across activity types. Developmental researchers argued that the influences of after-school time activity may vary due to the environment that fails to meet the adolescents’ developmental needs (7, 38, 39). Prior studies showed that poor family functioning was associated with adolescents’ problem and risky behaviors (25, 26, 29), poor school adjustment, and fewer positive developmental outcomes (35, 40, 41). Farb and Matjasko (42) noted that “adult supervision could be investigated as a moderator or mediator of participation with developmental outcomes” (p. 44). It seems that Chinese adolescents, who perceived higher family functioning, tend to receive better parental supervision and care, thereby likely to engage in more academic and structured activities than those who perceived lower level of family functioning. Along with this line, it is important to examine the characteristics of the activities (42, 43). As such, the current study will examine the relationship between family functioning and after-school time activity.

In light of the above considerations, the purpose of the present study was to assess the relationships between participation in after-school activity and personal well-being (i.e., positive youth development, risk behavior, life satisfaction, and school adjustment indicators) and family functioning among junior Chinese adolescents in Hong Kong. Three research questions were addressed in this paper:

1. What do Chinese adolescents do after school? What is the pattern of after-school activities engaged by the students?
2. Do adolescents engaging in different after-school activities have different levels of personal well-being indexed by positive youth development qualities, risk behavior, and perceived academic and school competence?

Based on the previous literature (35), it was expected that compared with those who participated in activities with peers and spent time alone, students who participated in after-school activities or spent times with adults would report higher levels of psychological well-being (i.e., positive youth development qualities, life satisfaction, Hypothesis 1a), school adjustment (i.e., perceived school and academic competence, Hypothesis 1b), and a lower level of risky behavior (Hypothesis 1c).

3. Do adolescents engaging in different after-school activities have different perceived family functioning?

Based on the prediction of family theories (e.g., family monitoring facilitates adolescent development) and previous research findings, it was predicted that adolescents participate in more structured activities after school would have better perceived family functioning than those participate in unstructured activities (Hypothesis 2).

## Materials and Methods

This study used data from the first-wave data of a 6-year longitudinal study, which attempted to examine the personal, social, and family well-being and developmental trends of adolescents in Hong Kong. In the study, a multi-stage cluster sampling method was used (44, 45). A total of 28 secondary schools were randomly selected based on the school list obtained from The Hong Kong Education Bureau (http://www.edb.gov.hk).

### Participants

A total of 3,328 Grade 7 students (mean age = 12.59 years, SD = 0.74), including 1,719 boys and 1,572 girls, were invited to complete a self-administered questionnaire. Thirty-seven students did not indicate their gender. The demographic information of the participants is summarized in Table 1.

**Table 1:** **Demographic information of the participants**.

	*n*	%
Gender
Male	1,719	52.2
Female	1,572	47.8
Place of birth
Hong Kong	2,590	78.3
Mainland China	655	19.8
Others	64	1.9
Parental marital status
First marriage	2,781	84.4
Divorced	209	6.3
Separated	73	2.2
Remarried	129	3.9
Others	104	3.2
Receiving financial aids
Yes	225	6.8
No	2,606	78.3
Others	465	13.9

### Instruments and procedures

Before the data collection, ethical approval from the Hong Kong Polytechnic University Review Board, parental/guardian, school, and participant consent was obtained. A standard protocol was used to ensure the surveys were administered uniformly by the research assistants. Students were informed that participation was voluntary and confidentiality of their responses was assured. During the data collection, students found no difficulties in understanding about the term “after-school activity.”

#### Personal well-being indicators

##### The Chinese Positive Youth Development Scale (CPYDS)

The 45-item short form of the Chinese Positive Youth Development Scale (CPYDS) is used to assess 15 positive youth development constructs, including bonding (e.g., *When I need help, I trust my parents will help me*), resilience (e.g., *When I face difficulty, I will not give up easily*), social competence (e.g., *I know how to communicate with others*), recognition for positive behavior (e.g., *When I complete my tasks, teachers will praise me*), emotional competence (e.g., *When I am unhappy, I can appropriately show my emotions*), cognitive competence (e.g., *I believe there is a solution for any problem*), behavioral competence (e.g., *I know how to say no to unfair requests*), moral competence (e.g., *I have high moral expectation about my behavior*), self-determination (e.g., *I am able to make wise choices*), self-efficacy (e.g., *I have little control of things that happen in my life*), clear and positive identity (e.g., *I can do things as good as others*), beliefs in the future (e.g., *I have confidence to solve my future problems*), prosocial involvement (e.g., *In this school, students are encouraged to have voluntary service*), prosocial norms (e.g., *I care about unfortunate people in the society*), and spirituality (e.g., *To me, life is very dull versus very exciting*). All subscales were measured by using a six-point Likert scale, except spirituality (i.e., seven-point Likert scale). Details of the items can be seen in Shek et al. (46, 47). A higher score indicated better positive youth development. This scale contains no reverse-scored items. Alpha of this scale in the study was 0.85 (mean inter-item correlation = 0.37).

##### Life satisfaction

Five items were used to assess the life satisfaction of the participants (e.g., *I am very satisfied with my living conditions*). A composite score was calculated to obtain the mean of the overall life satisfaction (48). Alpha of this scale was 0.85 (mean inter-item correlation = 0.55).

##### Perceived academic and school performance (ASC)

Three items were used to assess participants’ perceptions on their ASC (e.g., *Are you satisfied with your own present academic performance?*). A composite score was calculated by averaging all item scores in order to obtain the mean of the overall ASC. Alpha of this scale was 0.67 (mean inter-item correlation = 0.40).

##### Delinquent behavior

Participants were asked to report how often they engaged in 12 types of delinquent behavior (i.e., stealing, cheating, truancy, running away from home, damaging others’ properties, assault, having sexual intercourse with others, gang fighting, speaking foul language, staying outside the home overnight without parental consent, strong arming others, and trespassing) in the past year. Alpha of this scale was 0.70 (mean inter-item correlation = 0.25).

##### Substance use

Participants were asked to assess their frequency of using eight types of substance (i.e., alcohol, tobacco, ketamine, cannabis, cough mixture, organic solvent, pills such as ecstasy, and methaqualone) in the past 12 months. A composite score was calculated to obtain the mean of the overall substance use. Alpha of this scale was 0.50 (mean inter-item correlation = 0.35).

#### Family well-being indicator

##### The Chinese family assessment instrument (CFAI)

The nine-item short form of the Chinese family assessment instrument (CFAI) is used to assess three dimensions of family functioning, including mutuality (e.g., *Family members love each other*), communication (e.g., *Parents often talk to their children*), and harmony (e.g., *Family members get along well*). A five-point Likert scale is used to measure these subscales. A higher total score on the subscales showed a higher level of positive family functioning. The reliability and validity of the scale was shown in other publications (47, 49, 50). Alpha of this scale was 0.90 (mean inter-item correlation = 0.50).

#### After-school activity

Participants were asked to report how they usually spend their time after school by using a single-item indicator (i.e., 1 = returned home with the presence of adult; 2 = returned home without the presence of adult; 3 = returned home with the presence of siblings only; 4 = extracurricular activity; 5 = socializing with friends without the presence of adults; 6 = wandering the streets alone).

### Data analysis strategy

All analyses were performed using SPSS 19.0. Descriptive statistics were conducted to describe all variables. To analyze activity type in terms of psychosocial well-being, academic and school performance, and risky behaviors, a series of multivariate analysis of covariance (MANCOVA) followed by analysis of variance (ANOVA) was used to test the differences in activity type on the outcome measures. Gender was included in the above analyses as covariate. *Post hoc* analysis was performed to avoid an inflated Type I error by adjusting Bonferroni *p*-values (*p* < 0.01).

## Results

As shown in Table 2, 42% of early adolescents returned home under adult supervision, 24% participated in extracurricular activity, 13% stayed at home without the presence of adults, about 21% spent time with siblings and friends, less than 1% wandered the streets along. Compared to the findings in the West (51, 52), more Chinese adolescents participated in adult-supervised after-school activity.

**Table 2:** **The prevalence of the after-school time activities**.

	Overall (%)
Returned home with the presence of adults	42.3
Returned home without the presence of adults	12.5
Returned home with the presence of siblings	10.9
Participated in extracurricular activity	23.5
Socializing with friends without the presence of adults	9.9
Wandering the streets alone	0.9

Tables 3 and 4 present the differences in psychosocial well-being, ASC, and risky behaviors regarding the types of after-school activity. The covariate effect on gender was significant (*F*_(1, 1665)_ = 9.61, *p* < 0.01). There was a significant main effect of the type of activity on all outcome variables (*F*_(5, 1665)_ = 3.80, *p* < 0.01). Adolescents participated in extracurricular activity and stayed at home under adults’ supervision scored significantly higher levels in all positive youth development qualities, ASC, family functioning, and lower level in risk behaviors as compared to other types of after-school time activity. In general, participants in structured activities (i.e., staying at home with adults and participating in extracurricular activity) scored higher in positive youth development qualities, ASC, perceived family functioning, and lower in risky behavior (i.e., past substance use and delinquency) as compared to those who participated in unstructured activities (i.e., socializing with peers and wandering street alone). The effect size (partial eta squared) results were shown in Tables 3 and 4. In general, the effect sizes for all measured variables were small, ranging from 0.02 to 0.11 (53). In short, Hypotheses 1a, 1b, 1c, and 2 were supported.

**Table 3:** **Differences in psychological well-being by types of after-school time activity**.

Variables	Home with adults^a^	Home without adults^b^	Home with siblings only^c^	Extra-curricular activity^d^	Socializing with friends^e^	Wandering streets alone^f^	*F*	η^2^
	*M* (SD)	*M* (SD)	*M* (SD)	*M* (SD)	*M* (SD)	*M* (SD)		
BO	4.88^abef^ (0.77)	4.66^abdef^ (0.91)	4.71^cef^ (0.78)	4.91^abdef^ (0.73)	4.40^abcde^ (0.95)	3.80^abcdf^ (0.91)	16.66**	0.05
RE	4.82^abef^ (0.84)	4.50^abdf^ (0.92)	4.64^cf^ (0.85)	4.85^bdef^ (0.75)	4.33^acde^ (0.89)	3.74^abcdf^ (0.93)	17.55**	0.05
SC	4.84^acf^ (0.83)	4.76^f^ (0.89)	4.74 (0.81)	4.92^bcdf^ (0.77)	4.74 (0.90)	4.05^abcdf^ (1.06)	4.25**	0.01
PB	4.50^acef^ (0.90)	4.29^bd^ (1.01)	4.28^acd^ (0.93)	4.57^cbdef^ (0.85)	4.07^ade^ (1.06)	3.60^adf^ (1.07)	11.76**	0.03
EC	4.44^aef^ (0.88)	4.28^bef^ (0.91)	4.30^cef^ (0.84)	4.47^def^ (0.86)	3.99^abcde^ (1.04)	3.45^abcdf^ (1.00)	11.40**	0.03
CC	4.43^ae^ (0.86)	4.36 (0.88)	4.34 (0.86)	4.51^def^ (0.81)	4.10^ade^ (1.03)	3.76^df^ (1.09)	7.13**	0.02
BC	4.62^ae^ (0.76)	4.63^be^ (0.79)	4.56^c^ (0.78)	4.73^de^ (0.74)	4.34^abde^ (0.91)	3.79^abcd^ (1.11)	9.03**	0.03
MC	4.52^abe^ (0.82)	4.29^abde^ (0.92)	4.38^cde^ (0.83)	4.63^bcdef^ (0.85)	4.05^abcde^ (0.89)	3.83^df^ (1.26)	15.02**	0.04
SD	4.58^aef^ (0.82)	4.45^bf^ (0.96)	4.49^cf^(0.83)	4.62^def^ (0.84)	4.26^adef^ (0.92)	3.57^abcdef^ (1.16)	8.61**	0.03
SE	4.45^af^ (0.90)	4.32 (0.96)	4.30 (0.90)	4.49^df^ (0.84)	4.25 (0.83)	3.64^adf^ (1.25)	5.19**	0.02
SI	4.21^ae^ (0.96)	4.01^bd^ (1.10)	4.06 (1.05)	4.28^bde^ (0.95)	3.89^ade^ (1.09)	3.74 (1.23)	5.75**	0.02
BF	4.56^aef^ (0.98)	4.36^bdf^ (1.10)	4.51^cef^ (0.94)	4.65^bdef^ (0.93)	4.06^acde^ (1.04)	3.38^abcdf^ (1.04)	13.18**	0.04
PI	4.54^abe^ (0.94)	4.26^bd^ (1.07)	4.36^cde^ (0.97)	4.69^bcde^(0.86)	4.07^acde^ (1.13)	3.90 (1.00)	13.43**	0.04
PN	4.82^abef^ (0.83)	4.56^abde^ (0.91)	4.71^ce^ (0.87)	4.91^bdef^ (0.78)	4.29^abcde^ (1.04)	4.07^adf^ (0.98)	17.33**	0.05
SP^g^	5.38^abef^ (1.20)	4.88^abd^ (1.42)	5.14^cdef^ (1.28)	5.52^bcdef^ (1.09)	4.66^acde^ (1.46)	3.86^acdf^ (1.30)	19.84**	0.06
LS	4.18^abce^ (1.00)	3.80^abd^ (1.20)	3.90^ac^ (1.00)	4.13^bde^ (1.04)	3.62^ade^ (1.13)	3.49 (1.22)	12.12**	0.04

*BO, bonding; RE, resilience; SC, social competence; PB, recognition for positive behavior, EC, emotional competence; CC, cognitive competence; BC, behavioral competence; MC, moral competence; SD, self-determination; SE, self-efficacy; SI, clear and positive identity; BF, beliefs in the future; PI, prosocial involvement; PN, prosocial norms; SP, spirituality; LS, life satisfaction*.

**p < 0.01.

*^a,b,c,d,e,f^Means sharing the same superscript indicate significant *post hoc* differences in the Bonferroni test (*p* < 0.05)*.

*^g^This is a seven-point Likert scale*.

**Table 4:** **Differences in risky behaviors, family functioning, and perceived academic competence by types of after-school time activity**.

Variables	Home with adults^a^	Home without adults^b^	Home with siblings only^c^	Extra-curricular activity^d^	Socializing with friends^e^	Wandering streets alone^f^	*F*	η^2^
	*M* (SD)	*M* (SD)	*M* (SD)	*M*(SD)	*M* (SD)	*M* (SD)		
DRUG	0.05^aef^ (0.11)	0.08^bef^ (0.15)	0.07^cef^ (0.22)	0.07^def^ (0.15)	0.22^abcde^ (0.33)	0.22^abcdf^ (0.21)	29.27**	0.08
DE	0.28^ae^ (0.34)	0.37^be^ (0.42)	0.36^ce^ (0.35)	0.32^de^ (0.37)	0.76^abcde^ (0.73)	0.70^ade^ (0.49)	41.19**	0.11
CFAI	3.98^abef^ (0.75)	3.65^abdef^ (0.81)	3.80^cef^ (0.72)	3.88^bdef^ (0.78)	3.20^abcde^ (0.84)	2.78^abcdf^ (0.63)	33.91**	0.09
ASC	3.22^abef^ (0.63)	3.17^bef^ (0.64)	3.21^cef^ (0.61)	3.21^def^ (0.65)	2.94^abcde^ (0.71)	2.62^abcdf^ (0.96)	7.57**	0.02

*Drug, past substance use; DE, past delinquency behaviors; CFAI, Chinese family functioning; ASC, academic and school competence*.

**p < 0.01.

*^a,b,c,d,e,f^Means sharing the same superscript indicate significant *post hoc* differences in the Bonferroni test (*p* < 0.05)*.

## Discussion

The purposes of the current study were (a) to describe the pattern of after-school time activity participation among Chinese early adolescents and (b) to assess the relationships between participation in after-school activities, personal well-being (i.e., positive youth development, life satisfaction, school adjustment, and risk behavior), and family functioning. There were several unique features of the study. First, a large and representative sample was used in this study. Second, in view of the paucity of research in different Chinese contexts, the present study extends the existing literature. Third, validated measures of positive youth development, adolescent risk behavior, and family functioning were used. Finally, both personal and family well-being indicators were covered in this study.

A majority of the respondents reported that they usually returned home with the presence of adults. Participation in extracurricular activity was the second most prevalent activity, followed by staying at home with and without others. The results of the present study were consistent with Shek’s findings (35) using a similar sample (34.7%). These results are higher as compared to the study in Western contexts (51, 52). The high level of returning home under adult supervision might be related to the emphasis of scholastic achievement in Chinese contexts (54, 55). Private tutoring (e.g., hiring English teacher) is common in Chinese families. Chinese parents often ask their children to spend time working at home for school, such as doing homework, preparing quiz, and examinations in order to achieve school success. Also, it is a common cultural belief that kids will become delinquent if they hang out in the streets too often (56).

Another possible explanation can be operated in terms of familistic values (i.e., mutual support and hierarchical pattern of authority) in Chinese contexts (57). Spending time together with family members is an attribute of a happy and harmonious family (58, 59). It also relates to healthy family functioning and serves as a protective factor against Chinese adolescents’ suicide ideation (60, 61). This study demonstrates the cultural characteristics of participants should be considered when studying the pattern of adolescents’ after-school time activity.

In the present study, socializing with friends alone was the second least popular activity. Adolescence is a period of exploration, experimentation, and searching of identity (62–64). To achieve identity formation and foster positive peer relations, adolescents participate in leisure and recreational activities and aimlessly wandering the street in their daily lives. Given the younger age of the sample in the present study, it is expected that the prevalence of social activity increase over time as socializing increase with age during the adolescence (65, 66). This is further supported by longitudinal studies in which healthy use of after-school time is a good indicator of a positive developmental trajectory throughout adolescence, regardless of the Western (67, 68) and Chinese contexts (35). Future research should adopt a longitudinal design to study the change of after-school activity among Chinese adolescents.

Although the prevalence of after-school activity without supervision is low, researchers noted this specific group, especially those who wandered the streets alone, deserves attention (43). Evidence shows significant associations between unsupervised after-school activity and early involvement in poor academic performance, substance use, and sexual behavior (14, 20, 69). This is further supported in a longitudinal study by showing the lower rates of criminal arrests and reducing chance to spent time with deviant peers among youth who participated in at least one extracurricular activity (70). In particular, Shek (35) found that unsupervised after-school activities, such as home alone and socializing with friends, are associated with poor parent–child relational qualities and psychological well-being. Our study shows the positive relations between supervised after-school activities and Chinese adolescents’ developmental outcomes. Of course, how to encourage a balance between structured activities and allowing freedom for adolescents to grow is an issue to be considered.

The results give support to the hypotheses that students who participated in after-school activities or spent time under adult supervision reported high levels of personal well-being in terms of positive youth development qualities, life satisfaction, perceived school, family functioning and academic adjustment, and a lower level of problem behavior as compared to those who spent time with peers or alone. When adolescents feel connectedness with adults, they are more likely to feel secure about themselves, to have prosocial attitudes and behaviors and to achieve academic success (6, 21, 71). The present study demonstrates that the nature of activity may be an important factor when studying its influence on adolescent development. More research on studying the components of adolescent after-school activities is warranted.

Results also showed that family functioning was better in students who engaged in structured activities. Based on the predictions of different family functioning theories, family functioning such as family rules and parental supervision governs adolescent development. The present findings are in line with such predictions. It can be reasoned that positive family functioning would shape positive parenting expectations, which would eventually constitute family protective factors for adolescent development.

Theoretically speaking, adolescent development is the product of multidimensional and reciprocal interaction between an individual and his/her environment (4, 7). The present findings show the relationship between participation in after-school time activities and adolescents’ developmental outcomes. The results of the study support the critical role of the individual and contextual assets on adolescent development (20) and highlight the need for future research to consider the nature of after-school activities. Practically, this may serve as an indicator of adolescent development in terms of their personal and family well-being. Practitioners and educators can use this indicator when designing high quality and structured after-school youth programs that meet the individual needs and characteristics in the future.

There are several limitations of the study. The first is that we only used one item to assess the pattern of adolescents’ after-school time activity. Although this approach has been adopted in previous study (6), future research should incorporate other measurement tools (e.g., the Children’s Assessment of Participation and Enjoyment, 11) to help clarify the multiple dimensions (e.g., recreational activities, social activities, skill-based activities) and features (e.g., intensity, duration, breadth) of after-school time activity and its relative effects on adolescents’ development and behaviors.

A second limitation is that the data are cross-sectional. Causal direction of effects based on the current results cannot be made. Future research should examine the effects of after-school activity by adopting a longitudinal design. Another limitation is the use of self-report measures. Future research should examine the influence of social desirability bias. Third, the effect size was generally small in the present study. Perhaps, this may be related to the large sample size. To further assess the influence of after-school activity on adolescents’ developmental outcomes, more research in this area is needed. Finally, possible protective factors in mediating the relationship of activity participation and adolescents’ developmental outcomes are suggested to be included. Prior findings show that the relationship does vary by parental monitoring, peer group characteristics, and neighborhood safety (32, 51, 68, 69, 72–74). More research is warranted to replicate and extend the present findings in Chinese contexts.

In conclusion, the findings of the current study highlight the importance of providing organized and supervised after-school activity to early Chinese adolescents. The results show the relationship between after-school activity and developmental outcomes. This study sheds light on this area of research and extends the literature that is mostly derived from Western contexts.

## Conflict of Interest Statement

The authors declare that the research was conducted in the absence of any commercial or financial relationships that could be construed as a potential conflict of interest.
